# TMEM45 protein family – Ancient residents of the cell endomembrane

**DOI:** 10.3389/fmolb.2026.1751599

**Published:** 2026-03-25

**Authors:** Xiaoqian Zhang, Yanping Zhang, Zhenguo Zhai, Xietong Wang

**Affiliations:** 1 Key Laboratory of Maternal & Fetal Medicine of National Health Commission of China, Shandong Provincial Maternal and Child Healthcare Hospital Affiliated to Qingdao University, Jinan, China; 2 Department of Obstetrics and Gynaecology, Shandong Provincial Hospital Affiliated to Shandong First Medical University, Jinan, China; 3 School of Clinical and Basic Medicine, (Institute of Basic Medicine), Shandong First Medical University, (Shandong Academy of Medical Sciences), Jinan, China

**Keywords:** DUF716, ECM, endomembrane, endoplasmic reticulum, TMEM45A, TMEM45B

## Abstract

The Transmembrane protein 45 (TMEM45) family comprises multi-pass transmembrane proteins that harbor the ancient DUF716 domain and are predominantly localized to the endomembrane system (endoplasmic reticulum, Golgi apparatus). In mammals, TMEM45 members exhibit highly tissue-specific expression patterns and their functions are tightly linked to endomembrane activities. TMEM45A directly binds prolyl-4-hydroxylase (P4HA1) to modulate extracellular matrix (ECM) synthesis, thereby contributing to fibrosis and corneal disorders. TMEM45B participates in the Golgi processing and trafficking of nociceptive signaling molecules and also influences viral replication. Another paralog, TEDDM1, is implicated in sperm maturation. Expression of TMEM45 proteins is stringently regulated by upstream signaling cascades including TGF-β1/Smad, hypoxia/HIF-1α, calcium signaling, and JAK2/STAT3. In turn, these proteins serve as regulatory nodes that modulate downstream pathways such as Jagged1/Notch, Rho/ROCK, unfolded protein response (UPR), NF-κB, AKT/mTOR, Wnt/β-catenin, DNA-damage repair, and apoptosis. This review integrates current knowledge on the tissue distribution and upstream/downstream signaling networks of TMEM45 proteins to clarify endomembrane protein function and provide new perspectives on intracellular signal transduction mechanisms.

## Introduction

Transmembrane proteins account for approximately 30% of cellular proteins and have been linked to various aspects of cell membrane functionality (Marx et al., 2020). They are responsible for a range of activities including signal transduction and function execution. The transmembrane proteins 45 (TMEM45) protein family, characterized by its unique DUF716 domain, was initially identified and associated with antiviral activities in plants (Davies et al., 2007; Luo et al., 2024). More recently, members of this protein family have been found to play significant roles in extracellular matrix (ECM), epidermal development, and pain signal transmission, among other vital biological processes (Lan et al., 2020; Hayez et al., 2014).

This paper will systematically discuss the functions of the TMEM45 protein family in Cell endomembrane, elucidating from the classification and structural basis of the TMEM45A protein family to its expression regulation and related signal transduction pathways-aiming to provide new perspectives for functional studies of membrane proteins.

## Classification and nomenclature of the TMEM45 protein family

**FIGURE 1:**
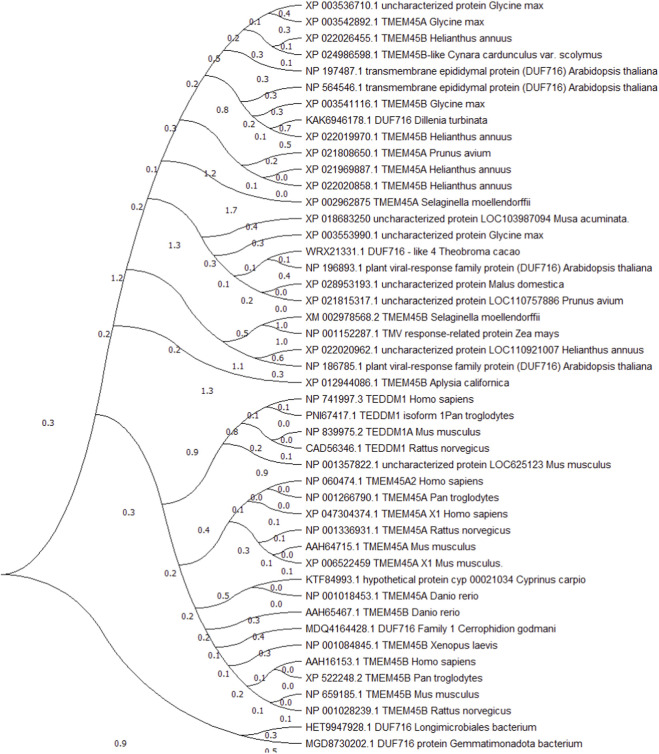
Phylogenetic analysis of the DUF716 protein domain across species. The evolutionary tree demonstrates that the DUF716 domain, which defines the TMEM45 protein family, is highly conserved from plants to mammals. Proteins from *Homo sapiens* (TMEM45A, TMEM45B, TEDDMI) are clustered with their orthologs in other vertebrates and are evolutionarily distinct from plant DUF716-containing proteins, highlighting the ancient origin of this protein family. Phylogenetic tree constructed using the neighbor-joining method in MEGA 11. Bootstrap values (≥70%) are indicated at internal nodes.

The TMEM45 family comprises ancient endomembrane multi-span proteins defined by the DUF716 domain (PFAM: PF04819). While DUF716-containing proteins are present in all three domains of life, the repertoire is markedly broader and more taxonomically widespread in Metazoa and Viridiplantae than in microbes, where the domain is sporadically retained in only a few phyla and with low paralogue number (Mistry et al., 2021). Phylogenetic reconstructions reveal shorter branch lengths among TMEM45 orthologues than between species, indicating that functional specialization of the family post-dates the divergence of the respective lineages (Figure 1; (Szollosi et al., 2015; Habib et al., 2024)). In mammals the DUF716 cohort has resolved into three distinct genes: *TMEM45A*, *TMEM45B* and *TEDDM1*.

**FIGURE 2:**
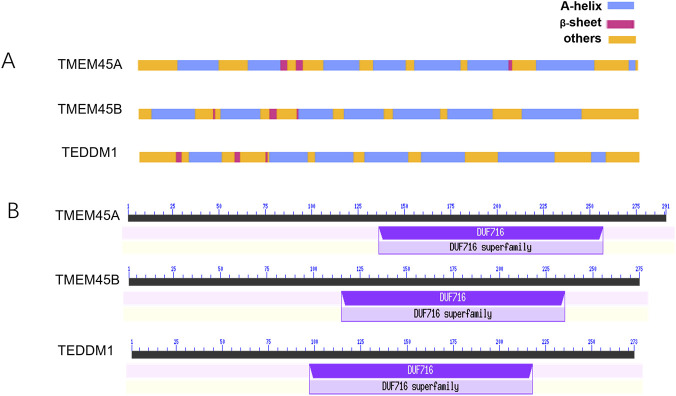
Analysis of the secondary structure and DUF716 domain in TMEM45s. **(A)** Predicted secondary structure elements (α-helices and β-sheets) for three representative TMEM45 protein members. The models reveal a conserved structural framework dominated by β-sheets and multiple transmembrane helices, suggesting a common molecular architecture and potential functional mechanism within the family. The topology is predicted using bioinformatics tools “TMHMM Server v2.0” **(B)** Mapping of the DUF716 domain (highlighted in red) onto the full-length protein sequence. The DUF716 domain is positioned as a conserved core within the protein, which strongly implies its critical role in the protein’s fundamental biochemical function.

*TMEM45A* (3q12.2) generates two alternatively spliced transcripts: *tmem45a1* (291 aa) and *tmem45a2* (275 aa), both enriched in skin, placenta and kidney. Secondary-structure prediction indicates eight α-helices and three β-sheets, with a seven-transmembrane topology (Figure 2; (Krogh et al., 2001; Kelley et al., 2015; Jumper et al., 2021)). *TMEM45B* (11q24.3; 275 aa) is predominantly expressed in stomach and small intestine; it is predicted to fold into seven α-helices, three β-sheets and seven membrane spans (Krogh et al., 2001; Kelley et al., 2015; Jumper et al., 2021; Okada et al., 2011). *TEDDM1* (1q25.3; 273 aa) is testis-restricted and adopts six predicted transmembrane helices, together with seven α-helices and three β-sheets (Krogh et al., 2001; Kelley et al., 2015; Jumper et al., 2021).

## TMEM45 structure and primary functions

Although catalogued within the TMEM superfamily, TMEM45 proteins exhibit profound structural and mechanistic divergence from both G-protein-coupled (e.g., TMEM87A) and ion-channel (e.g., TMEM16) paradigms (Zhang et al., 2020a; Galietta, 2009; Hoel et al., 2022). Their activity is embedded in endomembrane (including the endoplasmic reticulum and Golgi apparatus) protein synthesis, processing, and secretion, rather than in ligand reception or ion flux (Hayez et al., 2014; Wang et al., 2025; Hayez et al., 2016). TMEM45 members serve as architectural scaffolds that coordinate extracellular matrix (ECM) synthesis, assembly and release (Niu et al., 2024). A broad subset of TMEM proteins governs collagen traffic. TMEM131 partners with TRAPPC8 to build a TRAPP submodule that escorts procollagen from the ER to the Golgi (Sung et al., 2025); TMEM39A cooperates with COPII to accelerate collagen export (Zhang et al., 2021). TMEM45A adds a catalytic layer by binding the prolyl-4-hydroxylase α-1 subunit (P4HA1) within the ER lumen, thereby driving the hydroxylation and productive folding of procollagen chains (Niu et al., 2024).

**FIGURE 3:**
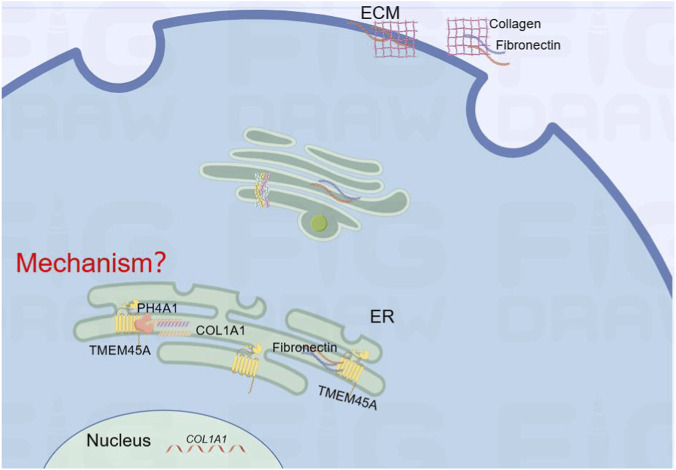
Putative role of TMEM45A in regulating extracellular matrix assembly. TMEM45A binds P4HA1, COL1A1, and fibronectin, suggesting a scaffolding role in ECM synthesis and assembly. However, how TMEM45A coordinates these processes remains unclear, as its molecular structure and detailed mechanism have yet to be elucidated (indicated by question marks).

By regulating ECM deposition, TMEM45A is identified as a factor of interest in organ fibrosis, with studies indicating its possible role as a network node (Xu et al., 2025a). Sustained TMEM45A expression is both necessary and sufficient for ECM over-production; siRNA- or CRISPR-mediated knock-down markedly reduces Collagen I, Collagen III and α-SMA during atrial fibrosis (Xu et al., 2025a). Besides, TMEM45A physically interacts with fibronectin and α-SMA, nucleating peripheral ECM assembly (Figure 3). Loss of TMEM45A triggers ECM insufficiency and keratoglobus (Weiner et al., 2021). Two pathogenic alleles illustrate the corneal phenotype: (i) exon 4 c.154C>T (p. Arg52) introduces a premature stop codon; (ii) the intron-5 acceptor mutation c.637G>T activates a cryptic donor within exon 4, deleting 50 residues that span a transmembrane helix. Both lesions reduce stromal collagen, leading to peripheral thinning, globular protrusion and lamellar disorganization.

TMEM45B is considered a relevant factor in nociceptive signaling in dorsal-root ganglia (Wang et al., 2025). Deletion impairs Golgi-dependent processing and anterograde axonal trafficking of pain-related peptides in IB4^+^ neurons, diminishing C-fiber synaptic input to superficial dorsal-horn laminae and blunting inflammatory mechanical hypersensitivity (Tanioku et al., 2022). Independently, TMEM45B binds Sindbis-virus RNA-capping enzyme Nsp1 and RNA-dependent RNA polymerase Nsp4, disrupting membrane-associated replication factories and sensitizing viral RNA to cytosolic RNases (Yan et al., 2022; Yan et al., 2024).

TEDDM1, the third DUF716-containing paralogue, is restricted to the initial segment of the epididymis in a steroid-independent manner. TEDDM1 participates in regulating post-testicular sperm maturation, but the underlying mechanism remains unclear (Yamazaki et al., 2006; Lin et al., 2017).

## Transcriptional regulation of the TMEM45 protein family

As polytopic transmembrane proteins that are excluded from the plasma membrane, TMEM45 paralogues are acutely sensitive to the physicochemical milieu of the endomembrane system. A convergent body of work shows that inflammatory cues, hypoxia and second-messenger pathways impose stringent, isoform-specific regulation on TMEM45 transcription and stability (Table 1) (Long et al., 2024).

**TABLE 1:** Upstream Regulators of TMEM45s.

Upstream signal	Cell type	Binding sequence	Related disease	Evidence levels
TGF-β1/Smad (TMEM45A)	Renal cells	5′GTGGGAA3′	Renal fibrosis (Zhang et al., 2021)	M [Rat ShRNA; Cell line]
HIF-1α (TMEM45A)	MDA-MB-231, HepG2	5’ACGTG'3′	Chemoresistance (Flamant et al., 2012; Luo et al., 2025)	A [Cell line siRNA; Bioinformatics]
Calcium signaling (TMEM45A)	Keratinocytes	N/A	Abnormal keratinization (Hayez et al., 2014)	M [Cell line]
JAK2/STAT3 (TMEM45B)	Gastric cancer	N/A	Gastric cancer (Zhao et al., 2016)	A [Cell line CRISPR]

## TGF-β1

Transforming growth factor-β1 (TGF-β1) is a pleiotropic cytokine whose dysregulation underpins fibrosis and cancer. TGF-β1 signaling has been identified as the dominant upstream axis for TMEM45A (Lan et al., 2020). In the 5/6-nephrectomy rat model, renal TMEM45A mRNA and protein rise in lockstep with endogenous TGF-β1. Exogenous recombinant TGF-β1 upregulates TMEM45A transcription and protein abundance in a dose-dependent manner in renal fibroblasts, hepatic stellate cells and atrial myofibroblasts.

### Hypoxia-inducible factor-1α (HIF-1α)

HIF-1α is the master transcriptional sensor of oxygen tension. TMEM45A is a direct HIF-1α target that links hypoxia to tumor progression, tissue fibrosis and chemo-resistance (Flamant et al., 2012). Two functionally validated hypoxia-response elements (HREs; 5′-RCGTG-3′) reside at −631/-627 and −218/-214 in the human TMEM45A promoter. Chromatin immunoprecipitation (ChIP) confirms HIF-1α occupancy at both sites under 1% O_2_; deletion of either HRE abolishes hypoxic induction. Pharmacological or siRNA-mediated HIF-1α silencing completely blocks TMEM45A upregulation in breast and liver cancer cells, restoring sensitivity to paclitaxel and etoposide. IL-10 fortifies this axis by activating JAK2/STAT3, which prolongs HIF-1α half-life and indirectly amplifies TMEM45A transcription. Beyond its role as a HIF-1α target, hypoxic migrasomes derived from colorectal cancer promote the expansion of Tmem45a^+^ fibroblasts and the expression of related genes (Luo et al., 2025).

### Ca^2+^ signaling

TMEM45A is a Ca^2+^-responsive gene in stratified squamous epithelia (Hayez et al., 2014). Stepwise elevation of extracellular Ca^2+^ (0.05 → 1.2 mM) triggers NFAT-driven TMEM45A transcription in keratinocytes, synchronizing the induction of early (KRT10) and late (involucrin, IVL) differentiation markers. In thymic Hassall’s corpuscles, Ca^2+^-dependent TMEM45A expression promotes terminal cornification and epidermal barrier formation.

### JAK2/STAT3

The JAK2/STAT3 cascade transduces inflammatory and oncogenic signals. TMEM45B is a direct transcriptional target of STAT3 (Zhao et al., 2016). IL-6 family cytokines (IL-6, LIF, OSM) activate JAK2, leading to STAT3-Y705 phosphorylation, nuclear translocation and occupancy of a conserved STAT3-binding motif (5′-TTCCCGGAA-3′) at −144/-136 in the TMEM45B promoter. CRISPR-Cas9 deletion of this element, or treatment with the STAT3-specific inhibitor static, abrogates cytokine-driven TMEM45B induction and attenuates downstream nociceptive and antiviral programs.

### Cell signaling networks orchestrated by the TMEM45 protein family

Although TMEM45 proteins are neither canonical surface receptors nor signaling kinases, their strategic residence in the endomembrane system enables them to gate multiple pathways indirectly-by modulating cargo trafficking, secretion or stability of transducers-thereby rewiring cell fate (Table 2).

**TABLE 2:** Signaling pathways associated with the TMEM45 family.

Pathway name	Biological effect	Associated disease	Evidence levels
Jagged1/Notch pathway (TMEM45A)	Promotes Jagged1 and Notch1 expression, leading to ECM deposition	Cardiac fibrosis (Xu et al., 2025a)	A [Rat; Cell line siRNA + OE; Human tissue]
Rho/ROCK pathway (TMEM45A)	Regulates RhoA and ROCK2 expression, affects cytoskeleton, promotes cell proliferation, adhesion, and invasion	Ovarian cancer metastasis (Guo et al., 2015)	P [Cell line siRNA]
UPR pathway (TMEM45A)	Upregulates UPR-related genes (MANF, PDIA6), enhances XBP1 splicing, inducing cells apoptosis	Cisplatin resistance (Schmit et al., 2019)	M [Cell line shRNA]
DNA damage repair pathway (TMEM45A)	Decreases EYA3 expression, impairs RAD51 recruitment to DNA damage sites	Cisplatin resistance (Schmit et al., 2019)	M [Cell line shRNA]
AKT/mTOR pathway (TMEM45A)	Sustain AKT and mTOR phosphorylation, activate the mTORC1 signaling	Palbociclib resistance (Chen et al., 2025)	P [Cell line siRNA]
NF-κB pathway (TMEM45A)	Upregulates NF-κB expression, promotes cell proliferation, migration and invasion	Glioma (Zhang et al., 2020b)	M [Human tissue Cell line siRNA]
TGF-β pathway (TMEM45A)	Activates TGF-β/Smad signaling, induces EMT and upregulates MDR1 expression, leading to chemoresistance	Colorectal cancer (Zhu et al., 2020)	M [Cell line siRNA]
Wnt/β-catenin pathway (TMEM45B)	Stabilizes β-catenin, upregulates downstream target genes (cyclin D1, c-Myc)	Osteosarcoma proliferation (Li et al., 2017)	M [Cell line siRNA]
Ca^2+^ signaling (TMEM45B)	Reduces ER Ca^2+^ storage capacity, impairs Ca^2+^-dependent signaling in DRG neurons	Chronic itch in atopic dermatitis/dry skin (Wang et al., 2025)	A [Mouse KO Cell line]
Apoptosis (TMEM45B)	Inhibits bax, promotes Bcl-2 expression, suppresses caspase-3 activity, reduces apoptosis	Inhibition of cancers cells apoptosis (Hu et al., 2016)	M [Cell line siRNA, OE]

ABB: A, advanced evidence; DRG, dorsal root ganglion; KO, knockout; M, mediate evidence; OE, overexpression; P, primary evidence; shRNA, short hairpin RNA; siRNA, small interfering RNA.

### TGF-β signaling

TGF-β not only induces TMEM45A but is itself subject to positive feedback from the protein. In 5-FU-resistant colorectal cancer cells, TMEM45A is upregulated and sustains TGF-β signaling to drive epithelial-to-mesenchymal transition (EMT) and multidrug resistance. Silencing TMEM45A downregulates TGF-β receptor I, blunts SMAD2/3 phosphorylation, reverts EMT markers (elevates E-cadherin, represses ZEB1 and vimentin) and suppresses the efflux pump MDR1 (Zhu et al., 2020).

### Jagged1/Notch signaling

Jagged1/Notch signaling represents a conserved juxtacrine mechanism that has been widely studied for its involvement in organ patterning. TMEM45A reactivates this axis in myofibroblasts, fuelling ECM over-deposition during injury repair and fibrosis. Over-expression of TMEM45A elevates Notch1 receptor and its ligand Jagged1, leading to accumulation of core ECM components. The γ-secretase inhibitor DAPT largely abrogates the pro-fibrotic output of TMEM45A over-expression (Lan et al., 2020).

### Rho/ROCK signaling

Rho/ROCK controls cytoskeletal architecture, motility and invasion. TMEM45A intersects with this pathway: its knock-down reduces RhoA and ROCK2 mRNA and protein in ovarian cancer cells and compromises proliferation, adhesion and invasiveness. Functional crosstalk between TMEM45A and TGF-β may jointly tune Rho/ROCK activity (Guo et al., 2015).

### Unfolded protein response (UPR)

As an integral membrane protein, TMEM45A contributes to ER proteostasis. Its depletion perturbs protein processing and folding, thereby modulating the UPR under stress conditions such as cisplatin exposure. Loss of TMEM45A upregulates UPR genes (MANF, PDIA6, HSP90) and augments XBP1 splicing; prolonged UPR dysregulation triggers apoptosis, although this effect is cell-context-dependent and is not observed in RCC4 renal carcinoma cells (Schmit et al., 2019).

### NF-κB signaling

NF-κB governs inflammation, survival and proliferation, and its chronic activation promotes gliomagenesis. TMEM45A positively regulates NF-κB: over-expression increases NF-κB mRNA and protein, whereas silencing has the opposite effect. siRNA-mediated NF-κB knock-down reverses the proliferative advantage conferred by TMEM45A over-expression in glioma cells (Zhang et al., 2020b).

### DNA-damage repair pathway

Homologous recombination is vital for genome integrity and cell survival. TMEM45A controls this process via the phosphatase EYA3. EYA3 dephosphorylates γH2AX (Tyr-142), facilitating recruitment of repair complexes (e.g., MDC1) and ensuring proper function of the recombinase RAD51. TMEM45A silencing downregulates EYA3 and cripples the repair trajectory (Schmit et al., 2019).

### AKT/mTOR signaling

AKT/mTOR integrates growth-factor and ECM cues to orchestrate anabolic survival. Suppression of TMEM45A markedly reduces p-AKT and p-mTOR levels in breast cancer cells and restores responsiveness to the CDK4/6 inhibitor palbociclib. The AKT/mTOR activator SC79 rescues the inhibitory phenotype, underscoring functional epistasis (Chen et al., 2025).

### Ca^2+^ signaling

TMEM45B integrates intracellular calcium homeostasis to orchestrate nonhistaminergic itch sensation in dorsal root ganglion neurons. Genetic deletion of Tmem45b markedly impairs calcium responses to β-alanine and allyl isothiocyanate in DRG neurons and alleviates chronic itch behavior in atopic dermatitis-like and dry skin-like mouse models. The reduced endoplasmic reticulum calcium storage capacity and downregulation of Serca1 following Tmem45b deficiency underscore functional epistasis, while pharmacological inhibition of Serca1 similarly suppresses intracellular calcium release, highlighting the critical role of TMEM45B-Serca1 axis in regulating ER calcium dynamics and itch transduction (Wang et al., 2025).

### Wnt/β-catenin signaling

Wnt/β-catenin controls proliferation, differentiation and stem-cell self-renewal; its aberrant activation is a hallmark of numerous cancers. TMEM45B appears to be involved in this cascade in osteosarcoma: its silencing is associated with reduced levels of β-catenin and relevant downstream targets, including cyclin D1 and c-Myc. As a Golgi-resident protein, TMEM45B may facilitate Frizzled maturation or shield β-catenin from the APC/Axin/GSK-3β destruction complex, thereby sustaining pathway activity (Li et al., 2017).

### Apoptotic signaling

Apoptosis maintains tissue homeostasis. TMEM45B governs this checkpoint in pancreatic, gastric and osteosarcoma cells. Silencing TMEM45B elevates the pro-apoptotic protein Bax, represses the anti-apoptotic Bcl-2 and activates executioner caspase-3. Conversely, TMEM45B over-expression reduces Annexin-V staining and enriches Bcl-2, favoring pancreatic cancer cell survival (Hu et al., 2016).

## Discussion

Emerging evidence indicates that the TMEM45 family may contribute to multiple biological contexts, spanning cell proliferation, differentiation, programmed cell death, as well as tissue repair, nociceptive signaling, and antiviral defense (Xu et al., 2025b). By physically and functionally interfacing with TGF-β, Notch, hypoxia-inducible factor (HIF) and the endoplasmic-reticulum unfolded protein response (UPR), these proteins govern tumor initiation, microenvironment remodeling, epidermal morphogenesis and fibrosis (Luo et al., 2025). Nevertheless, the precise molecular mode of action of TMEM45 proteins and their hierarchical position within complex signaling networks remain enigmatic. They are predicted to be multi-span transmembrane residents of the endomembrane system, yet whether they function as channels, transporters, scaffolds or enzymes is unknown (Shi et al., 2025). Direct experimental evidence is lacking for how they influence cargo trafficking, lipid metabolism or inter-organellar communication. The functional significance of the universally present but poorly characterized DUF716 domain, and the structural basis for the distinct biological outputs of highly paralogous family members, cannot be deciphered without high-resolution three-dimensional information.

Interestingly, the interaction between TMEM45A and signaling pathways exhibits pronounced context dependency. In head and neck squamous carcinoma SQD9 cells, TMEM45A silencing activates UPR (increased XBP1 splicing, upregulation of MANF, PDIA6, and HSP90B1) and enhances cisplatin-induced apoptosis. In contrast, the same intervention in renal carcinoma RCC4+pVHL cells fails to trigger apoptosis and instead confers cisplatin resistance. Similarly, TGF-β signaling exhibits cell-type specificity: in epithelial tumor cells, it drives EMT (downregulating E-cadherin, upregulating N-cadherin and Vimentin) and induces MDR1 expression to promote chemoresistance; in fibroblasts, it stimulates ECM component synthesis and inflammatory cytokine release, driving fibroblast-to-myofibroblast transformation and tissue fibrosis.

Notably, while the cited studies have established functional associations between TMEM45A and other signaling pathways, detailed and specific mechanistic investigations into its regulatory role remain limited. More importantly, the molecular structure of TMEM45A has not yet been elucidated. Therefore, further and more precise studies are warranted to comprehensively understand the functional mechanisms of TMEM45A.

## Outlook

### High-resolution structure and biochemical function

Determination of the atomic architecture of TMEM45 paralogues is the *sine qua non* for understanding their activity. AlphaFold 3 (or successor) models should be generated and iteratively refined against experimentally derived maps (cryo-EM, X-ray or micro-ED) to locate trans-membrane helices, cytosolic/luminal loops and putative ligand- or protein-interaction surfaces. Such structures will guide site-directed mutagenesis to test whether DUF716 constitutes a catalytic site, a lipid-binding pocket or a protein–protein interaction module.

### Wiring diagrams of TMEM45-centred signal transduction

Elucidating how TMEM45 proteins relay information across membranes is pivotal to explaining their impact on cell fate. Possible mechanisms include control of Ca^2+^ flux, regulation of membrane tension, or nucleation of signaling complexes at ER–Golgi contact sites. Affinity-purification mass spectrometry (AP-MS), proximity labelling (BioID, APEX) and single-cell multi-omics should be deployed to build comprehensive interactomes and time-resolved phospho-proteomic signatures (Jiang et al., 2021). CRISPR-Cas9 knockout or degron-mediated acute depletion followed by epistasis mapping will position TMEM45 within the TGF-β, Notch, Wnt and UPR hierarchies and clarify how they modulate myofibroblast differentiation, ECM secretion and therapy resistance (Lee et al., 2012).

### Clinical translation as diagnostic and prognostic biomarkers

Both TMEM45A and TMEM45B are markedly upregulated in multiple human malignancies and fibrotic disorders, offering untapped translational potential (Wen et al., 2024). Elevated TMEM45A correlates with histological grade, TNM stage and shortened overall survival in hepatocellular carcinoma, lung adenocarcinoma and head-and-neck squamous cell carcinoma (Sun et al., 2015), whereas TMEM45B is an emerging predictor of outcome in colorectal cancer, clear-cell renal carcinoma and prostate cancer (Paulo et al., 2012; Kuppuswamy et al., 2014). Prospective, multi-center cohort studies and standardized IHC or plasma-ELISA protocols are now required to validate the sensitivity, specificity and dynamic range of TMEM45 family members as biomarkers, and to evaluate their utility for early detection, therapy stratification or minimal-residual-disease monitoring.
